# Susac's Syndrome: A Tale of Disability Due to Late Recognition

**DOI:** 10.7759/cureus.74545

**Published:** 2024-11-26

**Authors:** Olusegun J Oluwole, Ane M Crespo Cuevas, Andrea Lorente Miranda, Vittorio Iantorno

**Affiliations:** 1 Neurology, King's College Hospital, Dubai, ARE

**Keywords:** brain-eye-ear syndromes, brain microinfarcts, brain white matter lesions, branch retinal artery occlusion, sudden sensorineural hearing loss (ssnhl), sudden vision loss, susac's syndrome

## Abstract

Susac's syndrome is a rare inflammatory microangiopathy characterized by the triad of retinopathy, encephalopathy, and hearing loss. The syndrome causes recurrent microinfarcts in these organs, which in turn manifests with repeated attacks of visual field loss, hearing loss and tinnitus, and various brain syndromes. These often lead to the significant accumulation of disability over time, particularly if there is a delay or failure in diagnosis. The brain lesions associated with this condition may resemble those of multiple sclerosis, especially to those who are unfamiliar with the disease and its distinctive clinical and radiological features. Such misdiagnosis may have grave clinical consequences. Here, we present the case of a 41-year-old Danish man who presented with the classical triad of Susac's syndrome but was misdiagnosed with multiple sclerosis. It took three years from disease onset before the eventual diagnosis was recognized, and by this time, he had accrued a lot of neurological deficits. With this case report, we aim to draw awareness to this rare but unforgiving entity which if missed can lead to the accumulation of disabilities. We also aim to emphasize the features that help distinguish it from multiple sclerosis, the condition with which it is most often confused.

## Introduction

In 1979, Dr. John O. Susac published the cases of two women with clinical presentations that posed diagnostic difficulties [1]. The first of the two women had presented with psychiatric manifestations, pyramidal signs, recurrent florid retinal infarcts, and disturbances of hearing and balance. The clue to the microangiopathic underpinning of the enigmatic presentation had come from ophthalmoscopic observations of multiple non-embolic branch retinal artery occlusions (BRAOs) as well as brain biopsy findings of sclerosis of small pial and cortical arteries. Investigations for all known causes of small vessel angiopathy were normal. The second patient had presented similarly but with more prominent attacks of acute hearing loss at onset. Both patients were eventually disabled by recurrent attacks involving the brain, retina, and ears. Susac's syndrome is an orphan disease with just about 500 cases published worldwide so far [2]. Its true incidence is believed to be probably higher, but many cases either are misdiagnosed as multiple sclerosis or go unreported.

This syndrome presents with a triad of encephalopathy, retinopathy, and sensorineural hearing loss (SNHL). Encephalopathy manifests acutely or subacutely with a range of symptoms that include headaches, focal neurological deficits, cognitive deficits, vigilance and mood disturbances, seizures, fatigue, and psychosis [2]. Hearing loss usually occurs suddenly due to cochlear microinfarcts, and there may be associated vestibular involvement. BRAOs account for visual loss, and the presentation is also sudden. Infrequent skin and gastrointestinal system involvement have also been described [2].

The underlying etiopathogenesis of Susac's syndrome is an immune attack on the endothelium of small arteries and arterioles of the brain, retina, and cochlea, and this process is believed to be driven by cytotoxic CD8+ cells [2]. Consequent endothelial cell hypertrophy and proliferation are believed to cause luminal obliteration and microinfarctions in the brain, cochlea, and retina [3,4]. Hence, the syndrome has been appropriately described by the acronyms "small infarctions of the cochlear, retina, and encephalic tissue (SICRET) syndrome" and "microangiopathy with retinopathy, encephalopathy, and deafness (M-RED)" [5]. Head MRI, retinal fluorescein angiography, and audiogram help to make the diagnosis. While head MRI shows brain infarcts typified by corpus callosal snowball lesions, fluorescein angiography shows BRAOs, and audiograms demonstrate low- to medium-frequency SNHL. The presence of all three elements of the triad qualifies for a definite diagnosis of Susac's syndrome, whereas the involvement of only two organs qualifies for a probable diagnosis. Most patients do not present with all three elements at first evaluation. In fact, those presenting with all three elements at the initial evaluation are said to be less than 20% of cases [6]. This could be an important cause of diagnostic delay, especially if Susac's syndrome is not considered in the differential diagnosis at the beginning [3,4]. Diagnostic forme frustes, such as isolated BRAOs and isolated cochlear infarcts, are also known to occur, further adding to the complexity of diagnosis and reporting [3]. There are no randomized controlled trials on the best treatments for Susac's syndrome. However, published literature supports the role of steroids, intravenous immune globulin (IVIg), immunosuppressants, and monoclonal antibodies [5]. Treatment guidelines have also been published by experts with considerable experience in the management of this condition [6].

Owing to the rarity of this condition and the high likelihood for misdiagnosis with grave implications, we present our case of a 41-year-old Danish man who presented over time with the classical triad of Susac's syndrome. His symptoms evolved over a period of 36 months and led to disability before the correct diagnosis was made. He had been misdiagnosed and treated as multiple sclerosis because of his brain lesions. His case will add to the existing literature on this rare entity and help increase awareness of its presenting features and how to distinguish it from mimics, the top among which is multiple sclerosis. Furthermore, the fact that the patient went into remission after receiving a single course of cladribine (offered with the intention to treat multiple sclerosis) raises an interesting question about the possible efficacy of cladribine in Susac's syndrome.

## Case presentation

**Figure 1:**
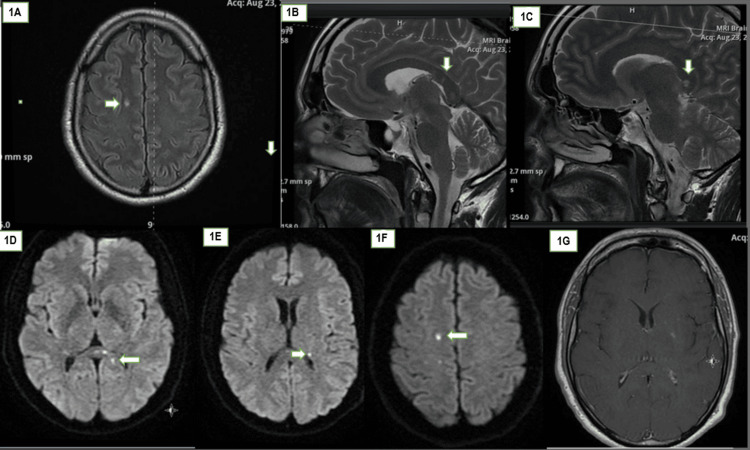
Brain MRI at the initial presentation in August 2020 (1A) A tiny FLAIR hyperintensity (arrow) in the deep white matter of the right hemisphere. (1B) A T2-weighted image showing a faint icicle lesion (arrow) in the posterior aspect of the corpus callosum. (1C) A T2-weighted image showing a small snowball lesion (a pathognomonic feature of Susac's syndrome) in the middle of the splenium of the corpus callosum. (1D) DWI with three tiny lesions in the splenium of the corpus callosum showing diffusion restriction. (1E and 1F) Other lesions showing diffusion restriction in the left and right hemisphere white matter, respectively. (1G) Post-contrast T1-weighted imaging showing a faint enhancement in the left internal capsule FLAIR: fluid-attenuated inversion recovery; DWI: diffusion-weighted imaging

A 41-year-old Danish man with a known history of hyperlipidemia on treatment with a statin was referred to the Neurology service by his endocrinologist in August 2020 on the account of orthostatic dizziness and random tingling in his face, lips, and fingers which had been present for several days. Prior to his referral, a head MRI scan had been obtained based upon a suspicion of multiple sclerosis. The MRI had revealed multiple white matter lesions which were reported as suggestive of multiple sclerosis (Figure 1).

Clinical assessment at this stage showed normal neurological examination, normal cardiovascular examination, and normal orthostatic vital signs. Owing to the sizes and distribution of brain lesions and the history of dyslipidemia, the assessing neurologist favored vascular etiology of the brain lesions and prescribed aspirin alongside advice to intensify lipid management. Dizziness was presumed to be caused by hypovolemia, and increased fluid intake was recommended.

About six weeks later, he returned with intolerable orthostatic dizziness, accompanied by decreased hearing and tinnitus. Clinical examination revealed significant orthostatic tachycardia upon standing, which was persistent and met the criteria for postural orthostatic tachycardia syndrome (POTS). Pure tone audiometry was normal despite subjective reduced hearing. He was managed for POTS with intravenous fluids and was discharged after 48 hours on non-pharmacological measures for POTS. On his follow-up visit one week later, he complained of dizziness upon rapid head turning and when negotiating roundabouts during driving, suggesting vestibular hypofunction. He also continued to experience muffled hearing in his left ear, along with tinnitus and hyperacusis. A petrous bone MRI was obtained by his ENT physician to exclude canal dehiscence, but it came back normal. All his symptoms slowly dissipated over the next five months.

**Figure 2:**
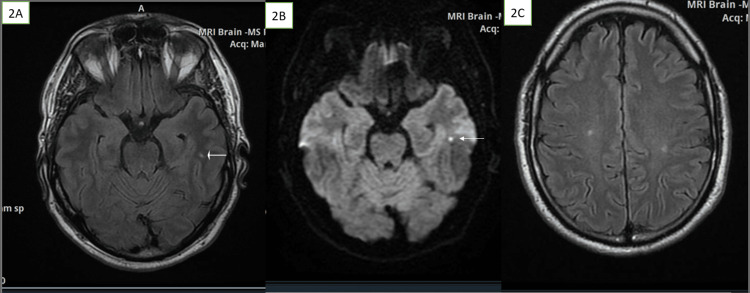
Brain MRI in March 2022 after the appearance of new symptoms (2A) A small new lesion in the left temporal lobe demonstrating a hyperintense signal on the FLAIR sequence (arrow). (2B) The same lesion showing diffusion restriction on the diffusion-weighted sequence. (2C) Some of the old lesions showing from August 2020, showing gliotic features on the FLAIR sequence FLAIR: fluid-attenuated inversion recovery

In July 2021, one year after the initial presentation, he developed an abnormal sensation in his feet, which felt like "walking on cotton wool." There was no objective sensory loss in his lower extremities except for allodynia at the soles of both feet. Numbness began within the next three days and slowly spread upwards to his perineum, together with "tiredness" in his calf muscles. Nerve conduction studies and somatosensory evoked potentials (SSEP) could not be performed at this stage because they were declined by the payer. He returned in March 2022 (eight months later) with spontaneous jerking in his legs, and his examination showed gait unsteadiness and lower extremity hyperreflexia. Head MRI was repeated and revealed new lesions which showed diffusion restriction in the white matter of the left temporal lobe. The old lesions from August 2020 were still visible but showed gliotic features (see Figure 2).

He continued to develop new symptoms, including sensory disturbances in both legs and the right hand as well as gait imbalance and tripping episodes. Whole spine MRI was normal (not shown). Physical therapy was prescribed at this stage. One month later, he presented to the ophthalmologist with two sequential episodes of sudden visual loss in his right temporal visual field. Right optic neuritis was suspected, and visual evoked potential (VEP) and ocular coherence tomography (OCT) were performed. VEP showed normal P100 latencies bilaterally, while OCT revealed increased retinal nerve fiber layer (RNFL) thickness in the right eye. Perimetry showed mild right upper field defect.

**Figure 3:**
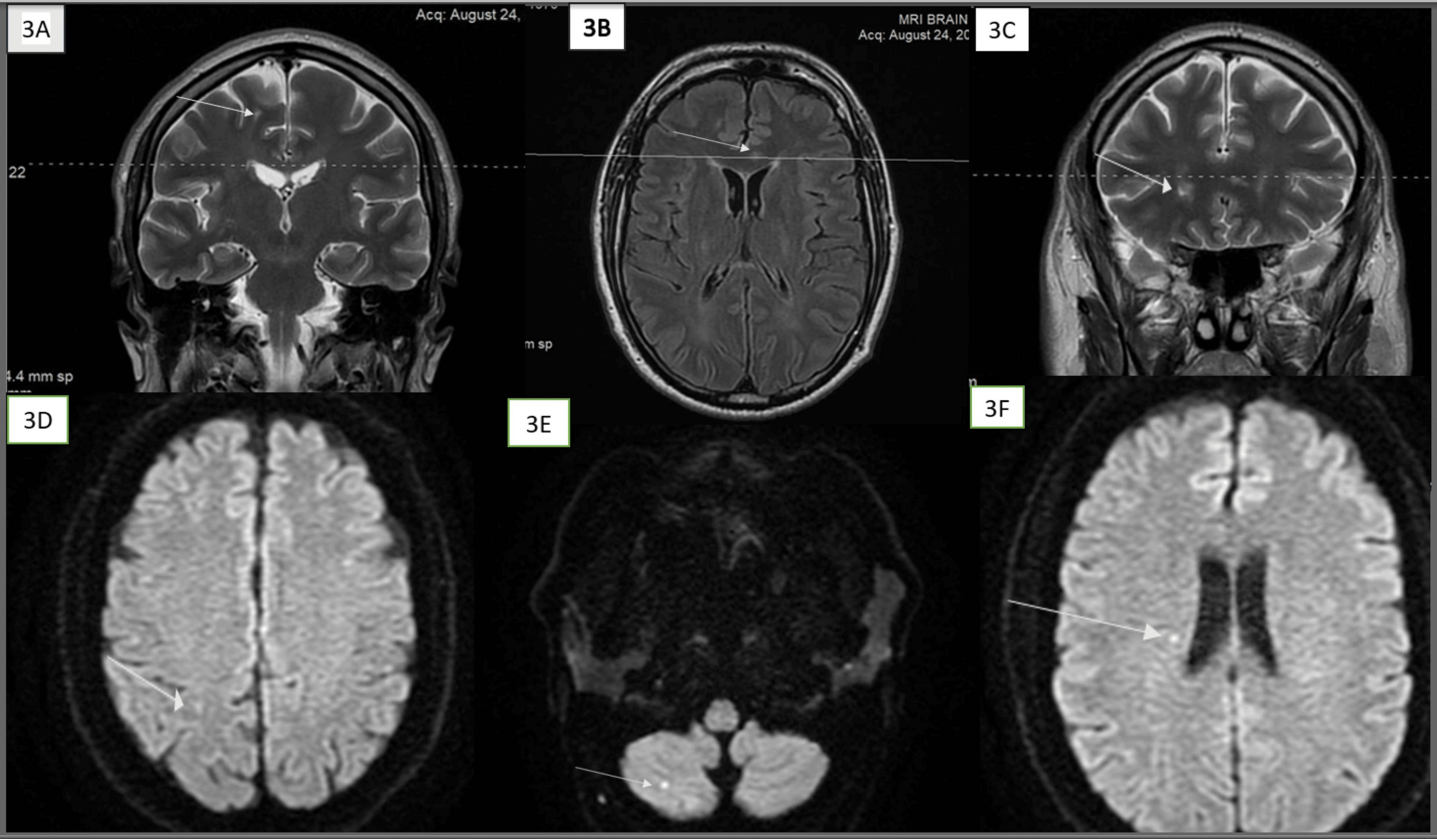
Brain MRI in August 2022 showing the development of a new set of lesions (3A) A new T2 hyperintense white matter lesion in the right superior frontal gyrus (arrow). (3B) Another new hyperintense lesion in the genu of the corpus callosum seen on the axial FLAIR image. (3C) The same image in 3B shown on T2-weighted coronal images. (3D) Another white matter lesion in the right parietal subcortical white matter, showing diffusion restriction. (3E) Yet another white matter lesion in the right cerebellar hemisphere showing diffusion restriction. (3F) One more new lesion in the right periventricular white matter showing diffusion restriction FLAIR: fluid-attenuated inversion recovery

In August 2022, he developed new-onset vertigo and blurring of vision and still had jelly legs and gait imbalance from his previous attack. Repeat head MRI showed new lesions in the right superior frontal gyrus subcortical white matter, genu and body of the corpus callosum, right corona radiata, right parietal subcortical white matter, and right cerebellar hemisphere (see Figure 3).

Within a week, he developed a new difficulty with micturition and urinary retention. His gait was ataxic, and he showed lower extremity hyperreflexia with bilateral ankle clonus. He was admitted for a five-day course of intravenous methylprednisolone, lumbar puncture, and in-patient physical therapy. His balance improved mildly after pulse steroid, but he continued to have sensory symptoms in his legs. Cerebrospinal fluid (CSF) analysis showed very mild mononuclear pleocytosis (6 WBCs/mm^3^), mildly increased protein (99 mg/dl), and the absence of oligoclonal bands. Tibial nerve SSEP and nerve conduction studies were performed at this stage and found to be normal. He showed modest gait improvement over the following month but continued to be bothered by dizziness and lower extremity cramps. At this stage, relapsing-remitting multiple sclerosis was seriously considered, and he was advised to repeat a head MRI in six months.

**Figure 4:**
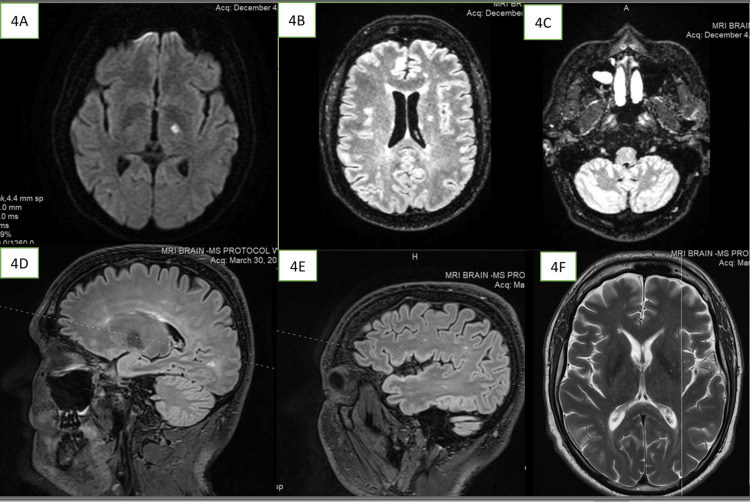
Brain MRI in December 2022 showing newly appearing lesions (4A) An obvious FLAIR hyperintense lesion in the left anterior thalamus. (4B) Multiple lesions in the periventricular and deep white matter showing diffusion restriction. (4C) One lesion in each of the cerebellar hemispheres showing diffusion restriction. (4D, 4E, and 4F) Multiple small lesions in the subcortical and deep white matter FLAIR: fluid-attenuated inversion recovery

Two months later, he made a new visit to the ENT physician for a fresh attack of sudden left-sided hearing loss and tinnitus. Pure tone audiometry and petrous bone CT scan were repeated. The petrous bone CT scan was normal, while the audiogram confirmed a new left SNHL in low frequencies. His hearing loss was significant this time and failed to improve despite a trial of oral prednisolone prescribed by the ENT. Hence, hearing aid was prescribed. Several weeks later, in December 2022, he was brought to the emergency room with a sudden onset of new brain symptoms, which included slurring of speech, dizziness, imbalance, and falls. Repeat head MRI showed gliosis of the old lesions as well as new lesions appearing in the anterior aspect of the left thalamus, frontoparietal subcortical white matter, periventricular white matter, and left cerebellar white matter. Some of the new lesions showed diffusion restriction but none showed gadolinium enhancement (see Figure 4).

The lesions were reported as likely demyelinating in nature because they were thought to not follow any vascular territories and to be oriented perpendicularly to the ventricles. An urgent admission was ordered for another five-day course of intravenous methylprednisolone following which he was discharged. He was lost to follow-up thereafter until June 2023. During this six-month gap, he had established care at another hospital where he was given a diagnosis of relapsing-remitting multiple sclerosis and started on disease-modifying therapy. He commenced the first course of oral cladribine at that hospital In January 2023 and completed it the following month. A few weeks after completing the first course of treatment, he developed sudden hearing loss in his right ear, followed within a week by further deterioration of hearing in his left ear. Shortly afterwards, he suffered an additional attack of sudden vision loss in the right upper quadrant of his visual field. He underwent a repeat head MRI at the end of March 2023 which showed numerous T2 hyperintense lesions in the juxtacortical and periventricular white matter, corpus callosum, and cerebellar hemispheres, with some lesions appearing hypointense on T1 (black holes), but there was no increase in lesion load in comparison to the immediate previous scan in December 2022.

**Figure 5:**
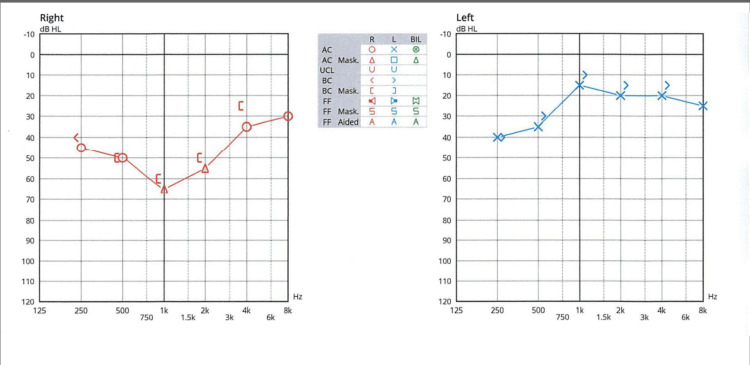
Pure tone audiogram in September 2023 after supposed clinical remission Notice the hearing loss in both ears, particularly in the lower frequencies

He came back to our hospital in June 2023. At the time, he had residues of urinary incontinence (for which he was taking mirabegron), gait unsteadiness, profound left SNHL, and right eye upper visual field loss. His neurological examination demonstrated lower extremity spasticity with hyperreflexia and gait ataxia. Bringing to the fore all of his different attacks over time, we considered the known brain-eye-ear (BEE) syndromes and came to the conclusion that the best fit which unified his complex symptomatology over the years was Susac's syndrome. Other diagnostic considerations were mitochondrial cytopathies, central nervous system (CNS) infections, primary and secondary CNS vasculitides, and genetic vasculopathies. Blood lactate was normal, CSF analysis was normal, serum inflammatory markers were normal, and vasculitis screening was also normal. Genetic tests for cerebral autosomal dominant arteriopathy with subcortical infarcts and leukoencephalopathy (CADASIL), cerebral autosomal recessive arteriopathy with subcortical infarcts and leukoencephalopathy (CARASIL), and TREX-1 mutation were also normal. A final diagnosis of Susac's syndrome was made in June 2023, nearly three years from disease onset. Considering that he had not developed any further new symptoms from March 2023 and that his brain MRI had been stable, we considered that he could be in remission. No further treatment was given, and he was placed on a six-monthly follow-up with audiometry, visual field assessment, and head MRI after due counseling regarding his diagnosis. One of his later audiograms done in September 2023 is shown in Figure 5.

Despite clear explanations about his diagnosis and a written plan to commence treatment at the earliest sign of future attacks, he disputed the diagnosis and went to another facility where his diagnosis was revised to aggressive relapsing-remitting multiple sclerosis. There, he was offered stem cell therapy. He underwent stem cell therapy in the first quarter of 2024. Despite stem cell therapy, his disease activity seemed to return in August 2024, when he experienced a new attack of BRAO in his right eye. He currently has gait impairment, hearing impairment, and visual impairment.

## Discussion

It took about three years from disease onset before our patient got the diagnosis of Susac's syndrome. This was despite the clear involvement of two organs (brain and ear) within the first year of disease onset, which would ordinarily have qualified him for probable Susac's syndrome [2]. It is an established fact that not more than 10-15% of patients show the classical triad at disease onset [3]. Hence, knowledge of this condition and a high index of suspicion are necessary to avoid misdiagnosis.

In retrospect, we think his earliest symptom complex of recurrent dizziness, hearing loss, and tinnitus within the first three months was likely a result of cochlear and vestibular organ microinfarcts which went unrecognized. One of the two original cases published by Dr. John O. Susac also featured prominent vestibular involvement [1]. Even though audiometry was normal early on, it eventually became abnormal after further hearing attacks and showed SNHL in low frequencies. This is consistent with the expectation in Susac's syndrome where low to middle frequencies are typically affected due to the preferential damage to the cochlear apex [3]. The fact that our patient eventually required a hearing aid points to the degree of disability that can occur when this condition is misdiagnosed or recognized late. Restricted presentations with isolated vestibular involvement have been reported, further emphasizing the need to have a high index of suspicion for this condition [5].

A wide array of symptoms can be expected to occur from cerebral involvement in Susac's syndrome. Headache is said to be the most common manifestation, seen in up to 80% of cases, while cognitive and psychiatric manifestations, confusion, focal neurological deficits, seizures, and fatigue are other common features [2,4]. Encephalopathy has been described in up to 75% of cases [4]. Our patient showed sensory disturbances early, followed by gait and balance difficulties, falls, dysarthria, and bladder symptoms. For unknown reasons, he did not experience headaches at any time and had no features of acute or subacute encephalopathy throughout his three-year course. We do not think this detracts from the diagnosis; it rather highlights the protean manifestations that can result from brain involvement in this syndrome. We also posit that cerebral involvement may result in the slow stepwise accumulation of focal neurological deficits over time with or without headaches and encephalopathy. This manner of presentation could be one of the reasons why this condition is often misdiagnosed as multiple sclerosis. Importantly however, there are established clinical and radiological distinguishing features between both entities. Sudden unilateral hearing loss is a rare occurrence in multiple sclerosis, occurring in less than 4% of patients, due to lesions affecting the brainstem or higher auditory pathways, and it would be expected to improve within a few weeks as do most typical symptoms of multiple sclerosis relapse [3,4]. On the contrary, our patient did not show brainstem lesions in the neighborhood of the cochlear nuclei. Hence, there was no reason to attribute his hearing loss to multiple sclerosis or to his brain lesions. Furthermore, bilateral tinnitus and hearing loss in multiple sclerosis are so rare they should be considered a red flag for an alternative diagnosis [4].

Retinal involvement in Susac's syndrome takes the form of BRAO and usually manifests with visual field defects [2]. Our patient experienced recurrent episodes of visual field loss confirmed on perimetry. Fundus fluorescein angiography (FFA) will usually demonstrate the BRAO and may also show areas of hyperfluorescence. Unfortunately, this test was not performed on our patient. In fact, his first attack of visual field loss was thought to be due to optic neuritis, hence the choice of VEP instead of FFA. Interestingly, VEP was normal in both his eyes and indicates that he did not have optic neuritis. Fundus photography, if done at the appropriate time, might also have shown retinal pallor, but this was omitted. The increased RNFL thickness reported in his OCT agrees with documented OCT findings in Susac's syndrome [5].

There are important radiological differences between the brain MRI appearances of Susac's syndrome and those of multiple sclerosis. Callosal involvement is common in both entities. However, while lesion location tends to be in the center of the corpus callosum (so-called snowball lesions) in Susac's syndrome, they tend to be at the periphery in multiple sclerosis [4]. Icicle and spoke lesions, hanging radially from the roof of the corpus callosum, are also unique to Susac's syndrome. In fact, some authors contend that the finding of these corpus callosal lesions in the context of encephalopathy is sufficient to make a firm diagnosis of Susac's syndrome even in the absence of SNHL and BRAO [7]. Another differentiating point is the shape of lesions which tends to be punctate in Susac's syndrome but ovoid in multiple sclerosis. Snowball lesions were present on our patient's images as shown in Figure 1. Other important differentiating features from multiple sclerosis are summarized in Table 1.

**Table 1:** Differentiating factors between Susac's syndrome and multiple sclerosis Reproduced and modified from Buzzard et al. [4] with kind written permission from Dr. Todd Hardy

	Susac's syndrome	Multiple sclerosis
Clinical		
Headache	Common	Uncommon
Encephalopathy	Common at outset	Uncommon at outset
Visual loss	Branch retinal artery occlusions	Optic neuritis
Hearing loss and tinnitus	Common	Uncommon
Focal neurological signs	Common	Common
MRI		
White matter lesions	Punctate	Ovoid
Corpus callosal involvement	Central (snowball lesions)	Peripheral
Infratentorial involvement	Common	Common
Meningeal enhancement	Common	Not present
Spinal cord disease	Rare	Common
Cerebrospinal fluid		
Elevated protein	Common	Uncommon
Mild lymphocytic pleocytosis	Common	Common
Unmatched oligoclonal bands	Uncommon	Common

Anti-endothelial cell antibody is reported to be positive in 20-30% of patients with Susac's syndrome [2,5]. It is however not specific for this condition as it may be found in other conditions as well [2]. This antibody was not tested in our patient because of its low sensitivity and specificity. Oligoclonal bands in CSF are only rarely reported in Susac's syndrome, another distinguishing feature from multiple sclerosis [2,3]. Not surprisingly, therefore, our patient showed absent oligoclonal bands in CSF. Primary CNS angiitis was excluded by normal intracranial MR angiogram and by involvement of the inner ear. Genetic microangiopathies of the CNS were similarly excluded by genetic testing, including CADASIL, CARASIL, and TREX-1 gene mutation.

Susac's syndrome is known to be steroid-responsive [1,2]. It is therefore not surprising that our patient showed improvements whenever he received pulsed methylprednisolone. The steroid treatments he received were however too short in comparison to what is ideal for inducing the remission of Susac's syndrome. Hence, we believe our patient was undertreated with steroids. Even though there are no randomized controlled trials about the most appropriate longer-term treatment for Susac's syndrome, there are anecdotal supports for IVIg, mycophenolate mofetil, cyclophosphamide, and natalizumab. Expert guidelines have also been published to guide the use of these agents in relation to the severity of disease [6]. After the diagnosis of Susac's syndrome was made, our patient was considered for azathioprine or mycophenolate, but we temporized on further treatment since he appeared to have gone into remission. The disabilities he accrued however remained, just as has been emphasized by other authors that Susac's syndrome can lead to significant disability if diagnosed late [5]. He continued to require a hearing aid and had enduring visual field losses, gait imbalance with spasticity, and bladder control difficulties. Even though Susac's syndrome can have variable clinical course, it has been shown that over 50% of patients followed beyond 24 months showed relapsing-remitting course [4,8]. We are not certain if our patient's initial remission was induced by cladribine's first course or the remission occurred naturally. It is however very plausible that cladribine induced the remission given that the latest retinal attack seemed to have happened slightly over 12 months from the initial course of cladribine, the exact period when the second course would have been due. This could be an indication that cladribine is potentially effective in controlling Susac's syndrome. It will be interesting to see whether future experiences from other centers will confirm our observation. Interestingly, accidental treatment of Susac's syndrome with multiple sclerosis medications may not always yield positive results, as there has been a report of exacerbation following treatment with natalizumab [9]. Therefore, it behooves neurologists to be aware of Susac's syndrome and its typical presenting features. An important lesson one can draw from this case, and indeed other cases in the literature, is that Susac's syndrome requires early diagnosis, timely treatment, and close monitoring in order to limit disability. Monitoring these patients could pose a difficult clinical challenge considering the wide array of symptoms that can occur and their complexity as well as the variability of clinical course and frequency of new clinical events among different patients. Inadequate monitoring would cause delayed or complete loss of recognition of new attacks which would translate to disability. Fortunately, Bullock et al. have recently created and published two clinical monitoring tools for Susac's syndrome patients, the Susac symptoms form (SuSx) and the Susac disease damage score (DDS), which can help patients and clinicians to adequately monitor patients' disease course, detect disability, and make timely and appropriate treatment decisions [10]. 

## Conclusions

Although Susac's syndrome is rare, it is crucial for the practicing neurologist to be aware of its clinical features and how it differs from multiple sclerosis. Our case illustrates the typical triad of Susac's syndrome and how late recognition can lead to disability. We also highlight the essential distinguishing features of Susac's syndrome from multiple sclerosis, the condition it is often confused with.
